# Lincoln estimates of mallard (*Anas platyrhynchos*) abundance in North America

**DOI:** 10.1002/ece3.906

**Published:** 2013-12-18

**Authors:** Ray T Alisauskas, Todd W Arnold, James O Leafloor, David L Otis, James S Sedinger

**Affiliations:** 1 Environment CanadaSaskatoon, Saskatchewan, Canada; 2 Department of Fisheries, Wildlife and Conservation Biology, University of MinnesotaSt. Paul., Minnesota; 3 Environment CanadaWinnipeg, Manitoba, Canada; 4 Department of Fish, Wildlife and Conservation Biology, Colorado State UniversityFort Collins, Colorado; 5 Department of Environment and Resource Sciences, University of NevadaReno, Nevada, USA

**Keywords:** band recovery, closed-population mark-recapture, harvest, population estimation

## Abstract

Estimates of range-wide abundance, harvest, and harvest rate are fundamental for sound inferences about the role of exploitation in the dynamics of free-ranging wildlife populations, but reliability of existing survey methods for abundance estimation is rarely assessed using alternative approaches. North American mallard populations have been surveyed each spring since 1955 using internationally coordinated aerial surveys, but population size can also be estimated with Lincoln's method using banding and harvest data. We estimated late summer population size of adult and juvenile male and female mallards in western, midcontinent, and eastern North America using Lincoln's method of dividing (i) total estimated harvest, 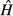, by estimated harvest rate, 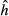, calculated as (ii) direct band recovery rate, 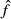, divided by the (iii) band reporting rate, 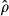. Our goal was to compare estimates based on Lincoln's method with traditional estimates based on aerial surveys. Lincoln estimates of adult males and females alive in the period June–September were 4.0 (range: 2.5–5.9), 1.8 (range: 0.6–3.0), and 1.8 (range: 1.3–2.7) times larger than respective aerial survey estimates for the western, midcontinent, and eastern mallard populations, and the two population estimates were only modestly correlated with each other (western: *r *=* *0.70, 1993–2011; midcontinent: *r *=* *0.54, 1961–2011; eastern: *r *=* *0.50, 1993–2011). Higher Lincoln estimates are predictable given that the geographic scope of inference from Lincoln estimates is the entire population range, whereas sampling frames for aerial surveys are incomplete. Although each estimation method has a number of important potential biases, our review suggests that underestimation of total population size by aerial surveys is the most likely explanation. In addition to providing measures of total abundance, Lincoln's method provides estimates of fecundity and population sex ratio and could be used in integrated population models to provide greater insights about population dynamics and management of North American mallards and most other harvested species.

## Introduction

Abundance of organisms is of fundamental interest to population ecologists and central to the study of many ecological processes. Many wildlife populations are harvested by humans, and population managers can prescribe levels of allowable take based on knowledge of population size, harvest effects, and carrying capacity (Runge et al. 2009). However, determining the number of individuals in free-ranging populations can be enormously challenging owing to difficulties in determining proper sampling frames, accounting for movement among subpopulations, and dealing with imperfect detection (Williams et al. 2002).

Since 1955, annual abundance of mallards and other North American ducks have been estimated using the Waterfowl Breeding Population and Habitat Survey (WBPHS), an internationally coordinated aerial survey conducted each spring, with accompanying counts by ground observers providing a visibility correction factor to account for birds not seen by aerial observers (Zimpfer et al. 2011). In addition, nearly 6 million mallards have been banded in North America since 1960, from which more than 1 million recoveries have been obtained. These data inform decisions about the interplay between exploitation rates and population size, and since 1995, annual allowable take of mallards in the United States has been governed by the concept of adaptive harvest management (Williams and Johnson 1995), wherein annual harvest regulations are based on estimates of annual population size and relative support for alternative models of density-dependence in fecundity and the degree of compensation for harvest mortality in survival (Nichols et al. 1995a).

Long before the WBPHS was adopted for monitoring duck populations in North America, Lincoln (1930) proposed that recovery of banded waterfowl could be used to estimate waterfowl population size if the total number of harvested waterfowl was also known. Lincoln's method for estimating waterfowl abundance from hunter recoveries was adapted and broadly applied to the capture–recapture of live animals and came to be known as the Lincoln-Petersen estimator (White and Bishop 2010). Lincoln's idea now forms the basis for most forms of closed-population capture–recapture estimators (Williams et al. 2002). Ironically, application of Lincoln's original method to estimate waterfowl abundance has been largely neglected and has seen limited use compared with its influence in capture–recapture studies (Pollock et al. 1990). Instead, the WBPHS continues to form the basis of monitoring programs for most species of dabbling and diving ducks in North America.

Bowers and Martin (1975) estimated range-wide abundance of wood ducks (*Aix sponsa*) using Lincoln's method, but this approach was not continued, despite absence of an alternative continental estimate of population size for this species. Boyd (1976); also Boyd et al. (1982) used Lincoln's method for snow geese (*Chen caerulescens*), but found that population estimates tended to be 50–100% higher than counts during mid-winter aerial surveys. Similarly, Otis (2006) applied Lincoln's estimator to mourning doves (*Zenaida macroura*) and midcontinent mallards, and although he found temporal parallelism between Lincoln and WBPHS estimates for mallards, Lincoln estimates were up to twice as high as WBPHS estimates. Alisauskas et al. (2009) examined Lincoln's approach for estimating abundance of four populations of arctic-nesting geese and obtained estimates that were 2–5 times larger than counts from traditional survey methods (see also Alisauskas et al. 2011). Our own unpublished analyses of northern pintails (*Anas acuta*), American black ducks (*Anas rubripes*), and American woodcock (*Scolopax minor*) have all resulted in population estimates much higher than those suggested by traditional survey approaches.

In this paper, we explore the utility of Lincoln's (1930) method for estimating abundance of mallards in three recognized North American populations, the western, midcontinent, and eastern stocks (U.S. Fish & Wildlife Service 2011). We predicted that Lincoln's estimates would be greater than WBPHS estimates in each case, in part because aerial survey coverage is incomplete for each population, but also based on our previous experience comparing Lincoln estimators to count-based surveys. The WBPHS represents a pre birth-pulse survey of adult mallards in May, whereas Lincoln's method represents a post birth-pulse survey that can provide estimates of the number of adult and young, males and females, that are alive during late summer banding efforts (Alisauskas et al. 2009).

## Methods

### Lincoln's estimator

Lincoln (1930) proposed that continental abundance of ducks could be estimated from knowledge about (i) the total number of birds harvested from a population, *H* and (ii) the proportion of the population that this harvest represents, that is, harvest rate, *h*. Considerable resources are invested in estimating both annual harvest and harvest rate of North American waterfowl. Annual harvest is estimated separately in Canada and the United States through questionnaire surveys of licensed hunters (Padding et al. 2006), and harvest rate is estimable from the direct recovery rate of banded birds, 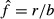, where *b* is the number of newly banded birds and *r* is the number of *b* that are shot during the first hunting season following banding, retrieved and reported to the Bird Banding Laboratory (Brownie et al. 1985). Typically, analysts assume zero mortality between banding and hunting periods, although *f* can be estimated even if this assumption is violated through concurrent modeling of survival rates (Brownie et al. 1985; Krementz et al. 2003), and we address this point with regard to Lincoln's estimator of abundance below. Since not all hunters report banded birds that they recover, the probability of band reporting, 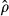, must be estimated separately (Henny and Burnham 1976), and applied to the probability of band recovery, 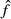, to arrive at an estimate of harvest rate, 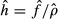. Thus, Lincoln's estimator is the quotient of two estimates,


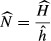
(1)

It is closely analogous to the Lincoln-Petersen capture–recapture estimator of abundance with population closure


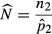
(2)

where *n*_2_ represents number of animals captured (i.e., harvested, 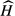, in the context of Lincoln's estimator) in the second of two sequential samples, and 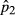 is the ratio of recaptures (i.e., number recovered, *r*) to animals marked in the first sample (i.e., number banded, *b*, or *n*_1_ in capture–recapture terminology). Thus, in the situation of a sample of uniquely marked individuals drawn from an exploited population of gamebirds for which estimates of harvest and band reporting probability are available, an estimator for population abundance at the time of banding becomes


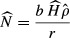
(3)

A bias-adjusted estimator, useful when sample size of *r* is small, was provided by Chapman (1951):


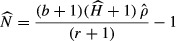
(4)

For simplicity, we have presented equations 1-4 without subscripts, but if harvest, banding totals, recovery totals, and reporting rates are recorded separately by year *i*, region *j*, age class *k,* and/or sex *l*, then abundance 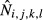 can also be estimated separately for each year, region, age class, and sex (Munro and Kimball 1982).

Ignoring probability of band reporting for the moment, an analogue of Seber's (1970) estimate of variance for Chapman's estimator would be


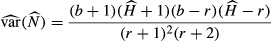
(5)

The estimated variance in the ratio of banding to recoveries is


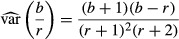
(6)

Applying the delta method for the variance of products, we estimated


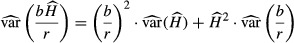
(7)

Finally, we arrive at an estimate of variance for the abundance estimate using the delta method for the variance of products:


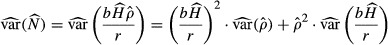
(8)

### Population delineation of mallards

**Figure 1:**
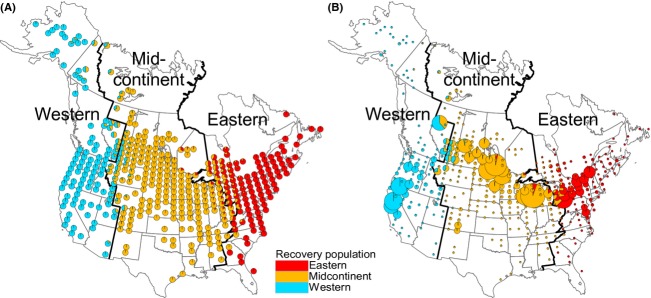
Western, midcontinent, and eastern mallard populations, as defined by location of capture and proportional recoveries. Pie diagrams in (A) denote the proportion of mallards banded in 1° latitude by 2° longitude quadrats that were recovered in western (blue), midcontinent (yellow), or eastern (red) populations in the subsequent hunting season. (B) Marking effort illustrated by symbols scaled according to number of mallards recovered from those marked in each quadrat; largest circle represents 9899 recovered mallards.

Adaptive harvest management (AHM) of North American mallards is focused on three defined stocks: western, midcontinent, and eastern (Fig. 1; U.S. Fish & Wildlife Service 2011). Aerial surveys conducted as part of the WBPHS, and additional state surveys, provide annual estimates of population size for each stock. Surveys for the western stock include mallards breeding in portions of Alaska, Oregon, and California, but exclude birds from most of Yukon Territory, British Columbia, Washington, Idaho, Utah, Nevada, and New Mexico. For purposes of AHM, the midcontinent stock is defined by the U.S. Fish & Wildlife Service (2011) as those mallards breeding in areas sampled by WBPHS strata 13-18, 20-50, and 75-77 (i.e., Northwest Territories, Alberta, Saskatchewan, Manitoba, western Ontario, Montana, North Dakota and South Dakota) as well as those breeding in Michigan, Minnesota, and Wisconsin and counted during state surveys. It is probably the most thoroughly surveyed stock, but survey coverage excludes Nunavut and portions of Manitoba and Northwest Territories, and mallards breeding in the central and southern portions of the Central and Mississippi Flyways are not enumerated. The eastern stock is defined by the U.S. Fish & Wildlife Service as those mallards breeding in a subset of 5 strata (51-54, 56 in southern Ontario and southern Quebec), as well as mallards breeding in the Atlantic Flyway states of Vermont, New Hampshire, Massachusetts, Connecticut, Rhode Island, New York, Pennsylvania, New Jersey, Maryland, Delaware, and Virginia that are surveyed by the Atlantic Flyway Breeding Waterfowl Survey (Heusmann and Sauer 2000). However, mallard populations have been expanding eastward during the last half century (Heusmann and Sauer 2000), and the population definition currently excludes large areas of potential breeding habitat in Maine, Quebec, and Eastern Canada. For comparative purposes, we included aerial survey data from Washington and Nevada in the western population, from Nebraska in the midcontinent population, and data from strata 51, 52, 63, 64, 66-68, and 70-72 were combined with the above-noted state survey data in the eastern population. These are the only strata in eastern Canada that have been surveyed consistently since 1990 (G. S. Zimmerman, personal communication).

### Harvest

Canadian and U.S. harvest estimates of mallards could be used in conjunction with band recoveries from both countries to estimate abundance (Alisauskas et al. 2009, 2011), but we elected to use only U.S. harvest estimates and recoveries for this analysis. Canadian harvest surveys began in 1969, whereas U.S. harvest surveys began in 1961, providing eight more years of data if we restricted our analysis to U.S. data only. Moreover, most harvest and recovery data (80–90%) are from the United States, so there was relatively small loss of information from basing our analysis on mallards harvested in the United States.

We acquired annual estimates of mallard harvest, 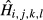 summarized by year *i*, flyway *j*, age class *k,* and sex *l* to correspond to the stocks of western (Pacific Flyway), midcontinent (Central and Mississippi Flyways), and eastern populations (Atlantic Flyway). Harvest data are partitioned into species, age class (hatch year, HY, vs. after hatch year, AHY), and sex based on a separate sample of wings submitted by hunters through the Parts Collection Survey (Padding et al. 2006). Importantly, age and sex ratios from the harvest data are known to be biased by differential vulnerability to harvest (typically, males more vulnerable than females, HY more vulnerable than AHY), but Lincoln estimates of population age and sex structure are derived from ratios of estimated cohort abundances and theoretically provide unbiased estimates of age and sex structure in the banded population (Munro and Kimball 1982).

Padding and Royle (2012) recently concluded that U.S. National Harvest Survey overestimates annual mallard harvest by 35–40%. It remains unknown if such a bias exists in Canadian harvest estimates – another reason that we restricted ourselves to using only U.S. harvest estimates in our analysis. Padding and Royle (2012) recommended that estimated U.S. mallard harvest from 1971 to 2010 should be multiplied by 0.73 ± 0.02 (95% CL) to eliminate this bias. Thus, we multiplied each 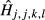 by 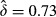 with 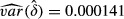.

### Banding and recoveries

We obtained banding records for all mallards marked with regular metal bands (i.e., excluding reward bands and any auxiliary markers) in Canada or the United States from 1 June to 30 September 1961–2011, and we obtained recovery records for any of these mallards that were reported as shot by hunters (BBL code HOW = 1) during the first hunting season following banding (1 Sept–15 Feb). We used all banding and recovery data to test assumptions about the regional fidelity of banded mallards to western, midcontinent, and eastern subpopulations. However, we restricted our analysis to U.S recoveries for Lincoln estimates so that recoveries would conform spatially to U.S.-based harvest estimates.

*Some assumptions* – The Lincoln estimator assumes that sampling is random, the population is closed, capture probabilities are independent, marks are not lost, marks do not influence survival, and all bands are reported by hunters (Seber 1982; Alisauskas et al. 2009). Random sampling, population closure, and band reporting are probably the most important assumptions (Alisauskas et al. 2009). Because Lincoln estimators are often applied to migratory game species, the closure assumption requires that harvest estimates are derived from the same population as are banding and band recovery data (Alisauskas et al. 2009). If birds move between populations between banding and harvest, it is a simple matter to exclude the marked birds that were recovered from another subpopulation based on their banding location, but there is no corresponding way to exclude such birds from estimates of total harvest. Nevertheless, if banded birds are a random sample of the total population, then movement rates of banded birds will provide an index of movement for unmarked birds. We assessed whether mallards from the western, midcontinent, and eastern stocks represented discrete populations by examining movement rates of marked birds between banding and harvest.

We defined populations based on origins of birds that were recovered in the Canadian provinces or states of U.S. Flyways as defined by the Bird Banding Laboratory (Fig. 1). For example, the border between Pacific and Central Flyways as defined by the Bird Banding Laboratory considers the states of Idaho, Utah, and Arizona to be contained entirely in the Pacific Flyway (FLYWAY CODE=4), and Montana, Wyoming, Colorado, and New Mexico as entirely in the Central Flyway (FLYWAY CODE=3). Thus, we defined the western stock based on origins of birds that were recovered in the Pacific Flyway states of Alaska, Washington, Oregon, California, Idaho, Nevada, Utah, and New Mexico, as well as British Columbia, Yukon Territory, and the southwest corner of Alberta (south of 56.6°N latitude, west of 113°W longitude) (Fig. 1). Midcontinent mallards included those recovered in the Northwest Territories, Nunavut, Alberta (north of 56.6°N latitude or east of 113°W longitude), Saskatchewan, Manitoba, Ontario (west of 86°W longitude), or any states in the Central (FLYWAY CODE=3) or Mississippi (FLYWAY CODE=2) Flyways. Eastern mallards included those that were recovered in Canada east of 86°W longitude or in any state of the Atlantic Flyway (FLYWAY CODE=1). We used a simple contingency table (PROC FREQ, SAS Institute. 2003) and SPANS GIS (PCI Geomatics. 2001) to summarize direct recoveries (whether recovered in western, midcontinent, or eastern populations), by their banding location grouped for every 1 degree of latitude and every 2 degrees of longitude (Fig. 1).

We used estimates of band reporting rates, 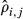 for each year *i* and subpopulation *j*, which could be applied to band recovery rates, 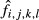, to estimate harvest rates for each year, population, age class *k,* and sex *l*:


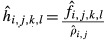
(9)

**Figure 2:**
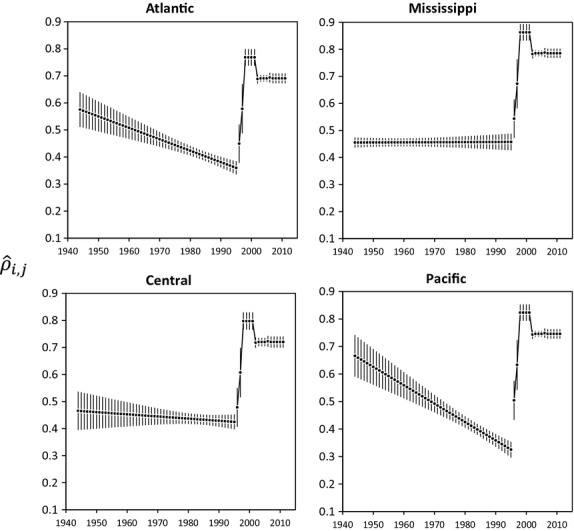
Probability,
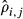
 ± 95% CL, that mallards recovered in year *i* and flyway *j* are reported to the Bird Banding Laboratory (R. Alisauskas, unpublished data). The pronounced increase during 1995–1997 was due to inclusion of toll-free telephone numbers on band inscriptions.

We used band reporting rates estimated from a concurrent meta-analysis of 13 published and unpublished studies of band reporting rates in North American waterfowl conducted between 1944 and 2010 (R. Alisauskas, unpublished data). Reporting rates were modeled according to year, whether bands had been solicited (i.e., reported to the BBL by agency personnel vs. by hunters themselves), how studies were designed (questionnaire vs. reward band), subfamily (ducks vs. geese), and the administrative flyway in which bands were recovered (Atlantic, Mississippi, Central, Pacific, and Canada). We did not consider age-or sex-specific reporting rates because there has been a paucity of estimates for juvenile and female mallards, although we acknowledge that Nichols et al. (1995b) provided evidence that reporting rates might be lower for female mallards. There was a pronounced increase in reporting rates beginning in 1995 when band inscriptions were altered to include a toll-free phone number (Royle and Garrettson 2005), and our models of annual band reporting rates therefore recognized a break point at this time (Fig. 2).

## Results

Of the 3,828,196 mallards banded from June to September 1961–2010, 20% were marked in the western, 60% in the midcontinent, and 20% in the eastern populations. Most western mallards (63%) were banded in California and Oregon, those from the midcontinent were banded primarily in Alberta, Saskatchewan, Manitoba, Minnesota, and Wisconsin, and those from the eastern population were banded most heavily in southern Ontario, southern Quebec, and Pennsylvania (Fig. 1B).

### Direct movement among populations

Of 271,129 mallards marked and directly recovered in Canada or the United States from 1961 to 2011 (Fig. 1A), 248,537 (91.7%) were recovered in the same geographic region (i.e., western, midcontinent, eastern) in which they were marked (Fig. 1A). The highest apparent regional fidelity was exhibited by western mallards (95.6%), followed by midcontinent (93.5%), and eastern mallards (86.1%). Movement was almost exclusively between adjacent populations, with the western population receiving birds from the midcontinent population (especially Alberta), and the midcontinent and eastern populations exchanging birds along their border (Fig. 1A).

### Harvest estimates, banding effort, and harvest rates

**Figure 3:**
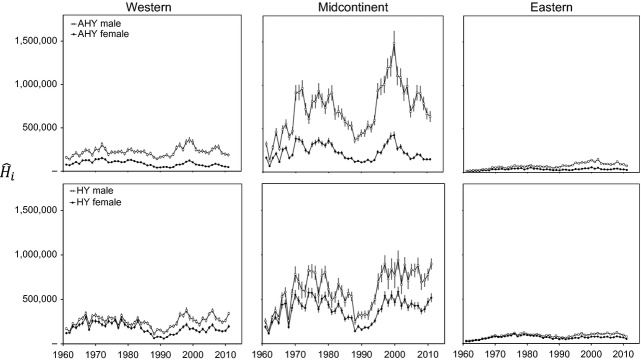
Estimated harvest of mallards in the United States, 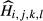
 ± 95% CL, in year *i*, 1961–2011, for population *j*, sex *k* (open symbols = males, closed symbols = females), and age *l* (AHY = after hatch year, top panels, and HY = hatch year, bottom panels). Harvest estimates were provided by R. Raftovich (U.S. Fish and Wildlife Service, unpublished data) and adjusted for bias as suggested by Padding and Royle (2012).

**Figure 4:**
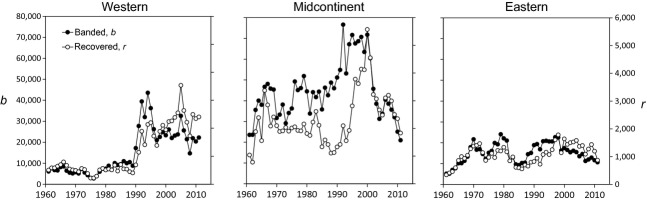
Number of mallards banded in Canada and the United States, *b* (left axes), and recovered in the United States, *r* (right axes), in three North American populations, as defined in Fig. 1. Note that axes are scaled so that parity represents a 0.075 recovery rate.

**Figure 5:**
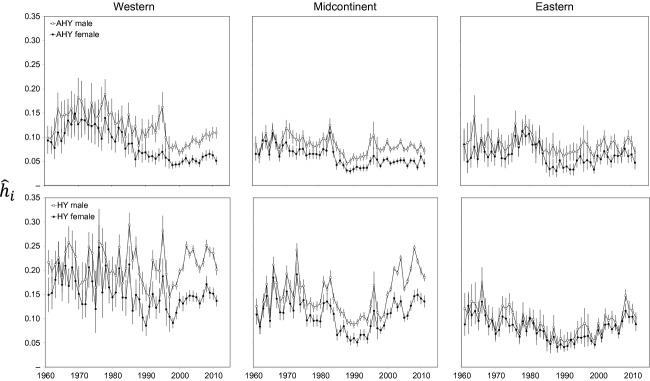
Harvest rate, 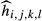
 ± 95% CL, of North American mallards in year i, 1961–2011, for population j, sex k (open symbols = males, closed symbols = females), and age l (AHY = after hatch year, top panels, and HY = hatch year, bottom panels). Estimates are based on rates of direct recovery in the United States only (Fig. 4) adjusted for band reporting probability (Fig. 2).

Numbers of mallards harvested each year in the United States were greatest in the midcontinent population, followed by the western and then eastern populations (Fig. 3). Harvest in the midcontinent population exhibited pronounced pulses associated with long-term wet cycles (e.g., 1970–1985, 1995–2011), but harvest was more stable in the western and eastern populations. However, all three populations exhibited pronounced increases in total harvest during the late 1990s. Number of bands applied in the western population increased markedly from ≤ 10,000/year before 1990 to ≥ 20,000/year afterward, whereas banding effort declined noticeably in the midcontinent during the last decade (Fig. 4). There were <25,000 mallards banded/year in the eastern population over the entire times series (Fig. 4). We estimated harvest rates, 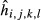 (Fig. 5), from these data by adjusting for estimated band reporting rates, 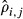 (Fig. 2). In all three populations, harvest rates were higher for males than for females, and higher for juveniles than for adults; harvest rates also tended to be higher for western mallards (AHY) than for midcontinent or eastern mallards (Fig. 5).

### Lincoln Estimates of Population Abundance

**Figure 6:**
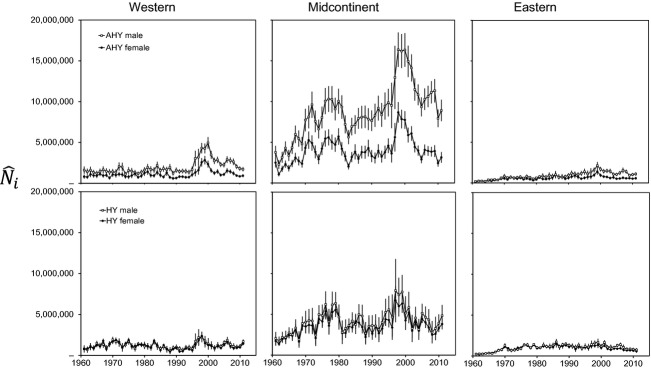
Lincoln estimates of abundance, 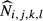
 ± 95% CL, of North American mallards in year *i*, 1961–2011, for population *j*, sex *k* (open symbols = males, closed symbols = females), and age *i* (AHY = after hatch year, top panels, and HY = hatch year, bottom panels).

Lincoln estimates (Fig. 6) closely tracked annual variation in number of mallards harvested from each respective population (Fig. 3). Lincoln estimates were most highly correlated with estimates of total harvest and band reporting rate (Table 1), although the magnitude of these correlations was consistent with that expected when one variable is also a component of the other variable involved in the correlation (Brett 2004).

**Table 1:** Correlations between Lincoln estimates of annual abundance, 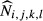, and estimates of total harvest, 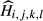, direct recovery rate, 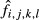, and band reporting rate, 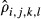, in year *i* (1961–2011), population *j* (western, midcontinent, and eastern), sex *k*, and age *i* (AHY = marked after the year of hatch, HY = marked the year of hatch).

Population	Cohort	Harvest (*H*)	Recovery (*f*)	Reporting (*ρ*)
Western	AHY F	0.30*	−0.16	0.61**
	AHY M	0.74**	−0.06	0.73**
	HY F	0.76**	0.02	0.31*
	HY M	0.85**	0.11	0.43**
Midcontinent	AHY F	0.74**	0.05	0.47**
	AHY M	0.88**	0.50**	0.71**
	HY F	0.65**	−0.05	0.32*
	HY M	0.70**	0.02	0.35*
Eastern	AHY F	0.54**	−0.34*	0.29*
	AHY M	0.91**	0.13	0.57**
	HY F	0.62**	−0.66**	−0.15
	HY M	0.64**	−0.54**	0.09

* *P *<* *0.05.

** *P *<* *0.01.

**Figure 7:**
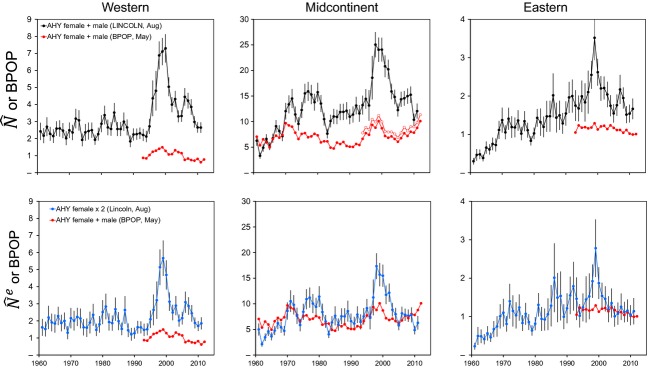
Lincoln estimates of abundance, in millions, of adult mallards in late August, 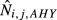
 ± 95% CL (top panels, black symbols and lines) and effective population size, 
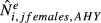
, ± 95% CL (bottom panels, blue symbols and lines) in relation to abundance in May, 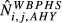, as estimated from aerial surveys during WBPHS (Zimpfer et al. 2011, closed red symbols and lines) in years 1961–2011. WBPHS estimates from Alaska were assigned from the midcontinent to western population. Also shown (open red symbols and lines) for midcontinent mallards are aggregate estimates from the traditional WBPHS survey and individual states (Michigan, Minnesota, Nebraska, and Wisconsin) from 1994 to 2012. Correlations between population estimates were 0.70, 0.54, and 0.50 for the top panels, and 0.70, 0.58, and 0.50 for the bottom panels, respectively.

**Figure 8:**
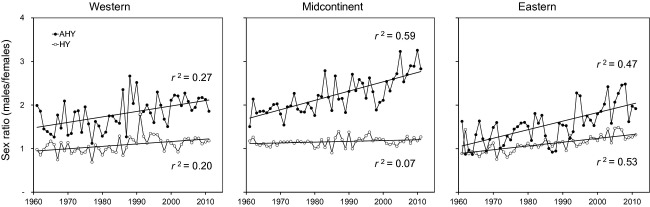
Sex ratios, 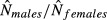 of North American mallards during 1961–2011 for each of three populations and each of two age classes (open symbols = hatch year, and closed symbols = after hatch year). Coefficients of determination, *r*^*2*^, for the linear change in age-specific sex ratios through time are shown near each regression line.

Lincoln estimates were only moderately correlated with WBPHS survey estimates (Fig. 7; *r*^2^ = 0.25 to 0.49). Lincoln estimates of adult population size in late August were higher than WBPHS estimates of the number of adult mallards alive in May in almost all cases (Fig. 7). The greatest discrepancy was for western mallards, where Lincoln estimates of adult population size averaged 4.0 (range: 2.5–5.9) times larger than WBPHS estimates. In the midcontinent region, Lincoln estimates averaged 1.8 (range: 0.6–3.0) times larger than WBPHS estimates. The midcontinent region had the longest time series of concurrent WBPHS estimates (60 years), and the two data sets exhibited strong concordance during the first 10 years, but diverged thereafter (Fig. 7). A similar concordance between Lincoln and WBPHS estimates was found for eastern mallards, where Lincoln estimates averaged 1.8 (range: 1.3–2.7) times larger than WBPHS estimates. The greatest difference between Lincoln and WBPHS estimates occurred during the late 1990s for each population (Fig. 7). Lincoln estimates expressed as effective population size, that is, which we define as two times the estimated number of adult females, were closer to WBPHS estimates: 2.7 (range: 1.7–4.1) for western, 1.1 (range: 0.4–2.0) for midcontinent, and 1.2 (range: 0.8–2.2) for eastern mallards. This suggests that part of the disparity between Lincoln and WBPHS estimates could be due to changes in population sex ratio. For all three populations, Lincoln estimates of adult sex ratios have become increasingly male biased through time (Fig. 8).

## Discussion

As noted in previous studies (Otis 2006; Alisauskas et al. 2009), Lincoln estimates of population size were consistently higher than concurrent estimates from count-based surveys. Moreover, Lincoln and count-based estimates of adult abundances in respective populations were not strongly correlated, suggesting that the 2 estimators are subject to different sources of sampling and/or estimation bias. We believe the most parsimonious explanation for this discrepancy is negative bias of existing survey protocols, and we emphasize four potential biases of existing survey methods.

First, large portions of each recognized breeding population were not surveyed using traditional count-based methods. This shortcoming is perhaps most notable for the western population, where current population indices are based on 12 relatively small WBPHS strata in Alaska and part of Yukon Territory, plus state surveys in California and Oregon, but ignore all of British Columbia, Washington, and Idaho, most of Yukon and Alaska, plus small but growing populations in Utah, Nevada, and Arizona (Sauer et al. 2011). Large portions of the range occupied by midcontinent and eastern populations also are unsampled during the WBPHS.

Second, count-based surveys provide an instantaneous sample for each survey stratum, but breeding birds may arrive over a protracted interval. Although survey chronology is opportunistically adjusted each year to account for annual variation in breeding chronology, constraints of surveying > 10 waterfowl species with variable migration chronologies inevitably leads to mismatched timing for at least some species. A recent study found that 18–44% of satellite telemetered northern pintails (*Anas acuta*) were outside of survey boundaries when aerial surveys were conducted (M. Miller, Western Ecological Center, U.S.G.S., unpublished data), indicating the potential for underestimation of true population size even if the entire breeding area is being sampled. For early nesting species like mallards, such timing issues are likely to lead to underestimates of the true breeding population via aerial surveys, but for late-migrant species such as lesser scaup (*Aythya affinis*), migrant individuals might be double counted (Afton and Anderson 2001).

Third, traditional count-based surveys such as the WBPHS may suffer from systematic underestimation of detection probabilities. Although WBPHS surveys include additional counts by ground-based observers designed to estimate detection probabilities for the aerial survey, the assumption that ground-based survey crews detect all available waterfowl is likely suspect. Pagano and Arnold (2009) found that ground-based observers detected 0.87 to 0.94 of available breeding pairs if observers were experienced, versus 0.54 to 0.89 of available pairs if they were inexperienced. In eastern Canada, detection probabilities are based on comparisons between fixed-wing aircraft and helicopters (Zimmerman et al. 2012), but the assumption that observers in helicopters see 100% of available birds has not been adequately tested.

Finally, the increasingly disparate sex ratio in favor of males, as inferred from Lincoln's method, may explain part of the discrepancy between Lincoln's estimates and those from the WBHPS, particularly in the midcontinent population. It is possible that methodology behind the WBHPS may fail to capture, or otherwise do not account for, the increasing number of superfluous males estimated with Lincoln's method.

Conversely, if Lincoln estimates are biased high, then either estimates of total harvest are biased high, or estimates of harvest rate are biased low. For all three populations, the greatest disparity between Lincoln and WBPHS estimates occurred during 1996–2001, a time period when methodology for estimating total harvest was revised (Padding et al. 2006), band inscriptions were changed to allow for electronic reporting (Royle and Garrettson 2005), and harvest regulations were liberalized in response to adaptive harvest management (U.S. Fish & Wildlife Service 2011); nevertheless, this was also a time period, at least within the midcontinent population, when traditional survey methods suggested record high mallard populations.

Underestimates of harvest rate could stem from overestimation of band reporting rates or from individual heterogeneity in recovery rates (Alisauskas et al. 2009). Band reporting rates could be underestimated if reward bands were not reported with a probability of 1, but many of the later estimates of band reporting rates we used employed $100 reward bands, for which previous analysis suggests that reporting probability approaches 1 (Nichols et al. 1995b; Royle and Garrettson 2005).

Lincoln estimates of population size were positively correlated with estimates of band reporting rate, but we believe this was a fortuitous correlation (i.e., band reporting rates and true population size were both substantially higher in the last 15 years), because WBPHS estimates of population size for the midcontinent population were also positively correlated with band reporting rates (*r *=* *0.47, *n *=* *51), and band reporting rates have nothing to do with aerial survey estimates. Heterogeneity in band recovery could also lead to overestimation of population size if banded birds are less vulnerable to hunters than unbanded birds. Such a scenario might apply if banding occurred primarily in refuge areas that were closed to hunting (as sometimes occurs with winter bandings; Blohm et al. 1987), but most discussions of potential bias in banding effort have postulated that banders are more likely to capture and band individuals that are more vulnerable to hunting (Weatherhead and Ankney 1984).

Padding and Royle (2012) recently demonstrated that U.S. harvest estimates were biased high, but we employed their correction factors in our estimates. Another potential bias would occur if estimates of total harvest included birds from outside the study population. Although we found that <10% of our banded sample of birds moved between populations, our samples of banded birds were not random (i.e., most of the banded sample in the western population was from Oregon and California, and these birds are primarily year-round residents). One potential solution to this problem would be to employ formal estimates of harvest derivation (Munro and Kimball 1982) for each flyway to adjust estimates of 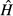 for the proportion of harvest that was derived from within each recognized subpopulation. Such analyses are ongoing (T. Arnold, unpublished data), but preliminary estimates of correction factors are 0.62 for the western population (which obtains a large portion of its total harvest from Alberta and other western locations within the midcontinent population), 0.95 for the midcontinent population (which obtains 5% of its harvest from the eastern population), and 0.87 for the eastern population (which obtains 13% of its harvest from the mid-continent population). Implementation of these correction factors would still result in Lincoln estimates that averaged ∼ 2.5-fold larger in the western population, 1.7-fold larger in the midcontinent population, and about 1.6-fold larger in the eastern population. We therefore conclude that the most likely explanation for this disparity is that WBPHS estimates are biased low.

We believe that Lincoln's (1930) method for estimation of animal abundance from exploited populations has enormous future potential for both population ecologists and wildlife managers. Theoretically, it could be used with any animal population beyond our example applied to 3 North American populations of mallards. The method could be extended to winter banding efforts of waterfowl, thereby obtaining estimates of abundance during late winter immediately after the hunting season (if demographic closure is violated between sampling periods, population estimates are still valid, but apply to the time of original marking; Alisauskas et al. 2009). For species such as mallards, this approach could give measures of population size before and after hunting and, in conjunction with measures of total harvest, could be used as an alternative method to assess the impact of harvest on population dynamics (U.S. Fish & Wildlife Service 2011). For species that are difficult to access or observe throughout much of their breeding range (e.g., American black ducks, lesser scaup, sea ducks), winter bandings might provide a more feasible monitoring method than aerial surveys during the breeding season (Alisauskas et al. 2009); however, harvest estimates are likely to be less precise for some of these species. Furthermore, Lincoln's method gives implicit measures of fecundity (i.e., fall age ratios) and sex ratios, and the banding data can also be used to estimate annual survival rates for each age and sex class (Brownie et al. 1985), thus providing all the necessary data to develop an integrated population model without ever counting birds (Otis 2006; Gauthier et al. 2007).

## Data Accessibility

Data available from the Dryad Digital Repository: http://doi.org/10.5061/dryad.m75g2.
